# Supplementary material for: NFATc2 enhances tumor-initiating phenotypes through the NFATc2/SOX2/ALDH axis in lung adenocarcinoma
Source: eLife. 2017 Jul 24;6:e26733. doi: 10.7554/eLife.26733 (PMC5570574; doi:10.7554/eLife.26733)
Supplement: Supplementary file 1. — DOI: http://dx.doi.org/10.7554/eLife.26733.030 [file elife-26733-supp1.docx]

**Supplementary file 1A. List of shRNA and gRNA sequences**

| **ShRNA sequences** | |
| --- | --- |
| **Sh-NFATc2-A** | CCGGCGCCAATAATGTCACCTCGAACTCGAGTTCGAGGTGACATTATTGGCGTTTTT |
| **Sh-NFATc2-B** | CCGGACGGAGCCCACGGATGAATATCTCGAGATATTCATCCGTGGGCTCCGTTTTTTG |
| **Sh-SOX2-A** | CGAGATAAACATGGCAATCAA |
| **Sh-SOX2-B** | CTGCCGAGAATCCATGTATAT |

| Oligos for NFATc2 gRNA cloning | |
| --- | --- |
| F: CACCGGGAGGCATTCGTGCGCCG  R: AAACCGGCGCACGAATGCCTCCC |  |

| Supplementary file 1B. List of PCR, qPCR, ChIP assay and cloning primers | | |
| --- | --- | --- |
| Primers for qPCR assay | | |
| *NANOG* | | F: AAGGTCCCGGTCAAGAAACAG  R: CTTCTGCGTCACACCATTGC |
| *SOX2* | | F: GCCGAGTGGAAACTTTTGTCG  R: GGCAGCGTGTACTTATCCTTCT |
| *OCT4* | | F: GGCAACCTGGAGAATTTGTT  R: GTGCATAGTCGCTGCTTGAT |
| *PPP3R1* | | F: CCTTTGGAAATGTGCTCACACT |
|  | | R: GGATTCTGTTGTAACTCAGGCAG |
| *CD166* | | F: ACTTGACGTACCTCAGAATCTCA |
|  | | R: CATCGTCGTACTGCACACTTT |
| *CD133* | | F: AGTCGGAAACTGGCAGATAGC |
|  | | R: GGTAGTGTTGTACTGGGCCAAT |
| *ALDH1A1* | | F: ATGCTTCCGAGAGGGGGCGA  R: CCCAACCTGGACAGTAGCGCA |
| *NFATc2* | | F: GAGGGGCTGTCAAAGCTCC  R: ACAGTTTTCCCCGTGATTCGG |
| *NFATc1* | | F: GCAGAGCACGGACAGCTATC  R: GGGCTTTCTCCACGAAAATGA |
| *PPP3R1* | | F: CCTTTGGAAATGTGCTCACACT  R: GGATTCTGTTGTAACTCAGGCAG |
| *RPL13A* | | F: GCCCTACGACAAGAAAAAGGG  R: TACTTCCAGCCAACCTCGTGA |
| *B2M* | | F: AGGCTATCCAGCGTACTCCA  R: GGCATCTTCAAACCTCCA T |
| Primers for cDNA amplification | | |
| *hNFATc2* | F: GCATCTAGAGCCACCCCCTACGAGGAAAGGGACCC  R: GCAGAATTCGCAGGAGGTCCTGAAAACTCC | |
| Primers for reporter construction | | |
| *SOX2*-luc-Region 1 | | F: CTGGGTACCCTTCAAACAATTATCCACAAGC  R: CTGGCTAGCTAACCTTCTTCCCATAATCACT |
| *SOX2*-luc-Region 2 | | F: CTGGGTACCTAAAATTACCCTCTTGGGTCCT  R: CTGGCTAGCTGGTGCAGGGTACTTAAATGAG |
| *SOX2*-luc-Region 3 | | F: CTGGGTACCAATTGCAAACTAGACATGCAA  R: CTGGCTAGCCCAGTACATATCTAAAACAAAGC |
| *SOX2*-luc-Region 4 | | F: CTGGGTACCGCTTTGTTTTAGATATGTACTGG  R: CTGGCTAGCCTCATCCTTTTGCACATTTC |
| *SOX2-*luc-Site 1 | | F: CTGGGTACCTAAAATTACCCTCTTGGGTCCT  R: CTGGCTAGCGAGTTCCCAGGACTCCAGCAG |
| *SOX2-*luc- Site 2 | | F: CTGGGTACCAAGGGCGTGAGAGAGTGTTG  R: CTGGCTAGCTAAATGAGGATGGGACGCGG |
| *SOX2-*luc- Site 3 | | F: CTGGGTACCAATTGCAAACTAGACATGCAA  R: CTGGCTAGCCTTTCCCTCTCCCCGAACAG |
| *SOX2-*luc- Site 4 | | F: CTG GGTACCTGCAAAACTCCCCGGCTTAT  R: CTGGCTAGCATATGGCTGTTGCCTGGCTT |
| *SOX2-*luc- Site 5 | | F: CTGGGTACCGGAAAGGGGTGGGGAAACAAG  R: CTGGCTAGCGTGATGCAGAAGCATCGCTA |
| *ALDH1A1-*luc- Site 1 | | F: CTGGGTACCGAATAACCCTACCTCTGTTCTATGT  R: CTGGCTAGCTTGAGAGAGTCAGCATTTCCTT |
| *ALDH1A1-*luc- Site 2 | | F: CTGGGTACCCCTGGCCTGGATTTGTCTTAT  R: CTGGCTAGCTGTCTGGACTTGAGTTGGTATG |
| Primes for ChIP-qPCR | | |
| *SOX2-* Site 4 | | F: ACTCCCCGGCTTATCATAAAC  R: TGCAGAGATTATCCGATTTGGG |
| *SOX2-* Site 5 | | F: TCCTCAGTGGGAGTGGAAA  R: GATGCAGAAGCATCGCTAAT |
